# Does sensory relearning improve tactile function after carpal tunnel decompression? A pragmatic, assessor-blinded, randomized clinical trial

**DOI:** 10.1177/1753193416657760

**Published:** 2016-07-08

**Authors:** C. Jerosch-Herold, J. Houghton, L. Miller, L. Shepstone

**Affiliations:** 1 School of Health Sciences, University of East Anglia, Norwich, UK; 2 Norwich Medical School, University of East Anglia, Norwich, UK

**Keywords:** Sensory relearning, cortical plasticity, nerve compression, sensory retraining, carpal tunnel syndrome

## Abstract

Despite surgery for carpal tunnel syndrome being effective in 80%–90% of cases, chronic numbness and hand disability can occur. The aim of this study was to investigate whether sensory relearning improves tactile discrimination and hand function after decompression. In a multi-centre, pragmatic, randomized, controlled trial, 104 patients were randomized to a sensory relearning (*n* = 52) or control (*n* = 52) group. A total of 93 patients completed a 12-week follow-up. Primary outcome was the shape-texture identification test at 6 weeks. Secondary outcomes were touch threshold, touch localization, dexterity and self-reported hand function. No significant group differences were seen for the primary outcome (Shape-Texture Identification) at 6 weeks or 12 weeks. Similarly, no significant group differences were observed on secondary outcomes, with the exception of self-reported hand function. A secondary complier-averaged-causal-effects analysis showed no statistically significant treatment effect on the primary outcome. Sensory relearning for tactile sensory and functional deficits after carpal tunnel decompression is not effective.

**Level of Evidence:** II

## Introduction

Sensory relearning (SR), also known as sensory retraining, is a behavioural intervention that makes use of ‘learning-dependent’ cortical plasticity. Graded tactile stimuli are used in combination with attention and intermittent use of visual feedback to facilitate improved tactile discrimination in the hand or upper limb (Jerosch-Herold, 2011). There is moderate evidence of its effectiveness in the treatment of functional sensory deficits in the hand secondary to peripheral nerve injury (Miller et al., 2012) and stroke (Carey et al., 2011; Schabrun and Hillier, 2009), as well as in the face following injury and surgery to the trigeminal nerve (Phillips et al., 2011).

Carpal tunnel syndrome is the most common entrapment neuropathy of the upper limb, characterized by signs and symptoms including pain, tingling, weakness and numbness in the distribution of the median nerve of the hand. The compression of the nerve leads to altered and decreased sensory input or, in severe cases, even a complete absence of sensation in the median nerve innervated territory of the hand (Middleton and Anakwe, 2014). This, in turn, causes maladaptive cortical changes in the somatotopy as demonstrated in functional magnetic resonance image (fMRI) studies (Maeda et al., 2013, 2014; Napadow et al., 2006). Although surgery can effectively alleviate symptoms of pain, tingling and numbness, those with severe and longer duration of symptoms are often left with chronic sensory disturbance (Middleton and Anakwe, 2014).

SR is not routinely offered to patients who have chronic sensory deficits after carpal tunnel decompression (Jerosch-Herold, 2011). To the best of our knowledge, only one pilot study has investigated the feasibility and acceptability of this intervention in carpal tunnel syndrome (Jerosch-Herold et al., 2012).

The objective of this definitive trial was to investigate whether a SR intervention taught as an unsupervised 6-week home therapy programme can improve tactile discrimination and self-reported hand function in patients with chronic sensory deficits after carpal tunnel decompression.

## Methods

### Study design and setting

This pragmatic, assessor-blinded, randomized controlled trial was conducted with the Norwich Clinical Trials Unit at the University of East Anglia. The term ‘pragmatic’ is used here in the context of a pragmatic trial (does an intervention work in those who are offered it?) as opposed to an ‘explanatory’ trial (does an intervention work under ideal conditions?) (Roland and Torgerson, 1998). Identification of potentially eligible patients took place in a large secondary care teaching hospital by searching the surgery records of the departments of Plastic & Reconstructive Surgery and Orthopaedics for patients who had carpal tunnel decompression at least 12 months ago or longer. The same method was also used in two primary care sites in Norfolk where carpal tunnel decompression is performed.

The screening, clinical assessment and subsequent randomization of eligible patients was conducted in four consecutive waves between February 2014 and February 2015. The National Research Ethics Service (REC reference 13/EE/0149) and local Research & Development departments at participating sites gave approval prior to study commencement. All patients gave written informed consent.

### Screening and selection

Screening for eligible patients was a two-stage process. First, all patients over 18 years of age who had undergone decompression surgery at least 12 months previously were sent an invitation to participate, including a participant information sheet and screening questionnaire. The screening questionnaire asked patients to rate any numbness (loss of feeling) in their hand (none = 1, mild = 2, moderate = 3, severe = 4 and very severe = 5) and any difficulty with grasping and use of small objects (no difficulty = 1, mild = 2, moderate = 3, severe = 4 and very severe difficulty = 5). Patients were asked which hand their responses referred to (left, right, both) and to indicate any other co-morbidities affecting their hand(s). Personal contact details and basic demographic information were requested to enable contact by the trial research staff.

Second, those indicating at least mild numbness and difficulty (score ⩾2 in each) in a previously operated hand were invited to attend the Norwich Clinical Trials Unit for a clinical assessment using validated sensory tests. Patients with sensory impairment secondary to other known aetiologies, such as stroke, were excluded.

Sensory impairment of the affected hand was assessed using standardized tests of sensibility, which are responsive in patients undergoing carpal tunnel surgery (Jerosch-Herold et al., 2011). Where patients had undergone bilateral decompression, the worst hand was assessed. Where both hands had similar levels of sensory impairment, the dominant hand was chosen. The tests assessed three aspects of sensory function: touch threshold (Weinstein Enhanced Sensory Test (WEST)), area localization (locognosia test) (Jerosch-Herold et al., 2006) and tactile gnosis (Shape-Texture Identification (STI^TM^) test) (Rosen and Lundborg, 1998). All patients gave written informed consent prior to the baseline assessment. Patients with scores below normal on at least two out of the three clinical sensory tests were eligible to participate in the trial and issued with a further patient information sheet and consent form.

### Randomization

Stratified blocked randomization with randomly permuted block lengths of two, four and six were used. The baseline tactile gnosis score (STI test) was used to stratify randomization by low (0–3 points) and high (4–6 points) STI score. The randomized sequence was generated by the data manager of the Norwich Clinical Trials Unit and held separately from the main study database.

Patients who were eligible for the trial following baseline assessment were seen by the Chief Investigator (CI) (first author) who answered any questions about the trial and obtained written consent to participate before completing two further baseline measures: the Moberg Pick-up test (Moberg, 1991) and three subscales of the Michigan Hand Questionnaire (MHQ) (overall hand function, activities of daily living (ADL) of affected hand and both hands, and work) (Chung et al., 1998).

### Blinding

Blinding of patients was not feasible in this trial. The allocation sequence was masked from the CI involved in randomizing patients to the trial (through online randomization). Both research associates involved in baseline and follow-up assessments were blind to group allocation. All randomized patients received verbal and written instructions not to divulge their group allocation to the assessor at both follow-up assessments.

### Intervention – SR

The SR programme was developed for the trial, based largely on a programme used in a previous pilot trial (Jerosch-Herold et al., 2012), and was designed to be practised daily at home. Using the principles of learning-dependent cortical plasticity, the SR programme proposes short intensive periods of tactile stimulation with and without vision, combined with cognitive learning techniques, to improve sensibility and function of the hand.

A standardized set of SR materials, as well as a booklet with written instructions and colour photographs, were given to patients. The CI explained and demonstrated the three SR exercises to patients emphasizing that these needed to be practised in a quiet environment for short periods (5–10 min) at least three times a day over 6 weeks. Patients were advised that they needed to modify the training materials to ensure that the exercises remained challenging, for example by incorporating smaller, more complex objects or relying less on vision and more on touch alone. It was emphasized that vision should be used only intermittently as feedback when identification by touch alone could not be accomplished. The first exercise used a 30 mm wooden cube with four different grades of sandpaper to be identified in order of smoothest to roughest and a 20 mm playing dice with raised dots from one to six. The playing dice was designed to be carried in a trouser or coat pocket to facilitate frequent daily practice. For the second exercise, an opaque cloth bag containing 14 small everyday items such as coins, buttons and screws was used. Patients had to identify each object by touch and without vision while attending to the feel of shape, weight, texture and form. For the third exercise, patients were asked to choose usual daily activities involving fine dextrous finger movement in which they needed to attend to the feeling of the shape, weight or texture of the items they were touching and using visual feedback only intermittently.

### Control group

Patients in the control group were instructed to use their hands as they had before. At the end of their trial participation they were offered written instructions on SR, including a description of materials that can be found around the house (e.g. assorted nuts, screws, buttons, textured fabrics) and how to practise the SR programme.

### Trial outcomes

All patients were assessed prior to randomization (baseline), at 6 weeks (end of treatment) and 12 weeks (end of follow-up). Each assessment comprised, in order of administration: (i) touch threshold (WEST) test; (ii) locognosia test; (iii) tactile gnosis (STI) test; (iv) Moberg Pick-up test; and (v) three sub-scales of the standardized MHQ. The methods of administration and scoring for the WEST, locognosia and STI tests have been described previously (Jerosch-Herold et al., 2011). Patient-perceived ability to use the hand in everyday activities and work was captured by three subscales of the validated MHQ.

The primary outcome was the tactile gnosis score assessed by the STI at 6 weeks. Secondary outcomes were the STI score at 12 weeks, touch threshold, locognosia test and Moberg Pick-up test and patient-reported hand function (MHQ).

Data on adherence to the home SR programme were collected through a patient completed daily diary in which patients were asked each day to indicate which exercises were completed, how often and for how long. The diary had 42 days (6 weeks) of rows to complete. At the end of the 6 weeks patients were asked to indicate if they continued with the exercises and, if so, why. They were also asked to comment on what they liked and disliked about the SR programme.

### Sample size

The sample size calculation was based on data from a previous pilot trial (Jerosch-Herold et al., 2012), which observed a pooled standard deviation for the STI of 1.605. Using a 1.09 point change observed in STI score over 4 months in a group of 63 patients undergoing surgery and who considered themselves improved on a global rating scale (Jerosch-Herold et al., 2011) as being clinically relevant, this provides an effect size of 0.68 (i.e. 1.09/1.605). Based upon this effect size (0.68), a power of 90% and 5% significance level, 47 patients per group would be required. Allowing for a 25% loss to follow-up a total of 126 randomized patients was aimed for.

### Statistical analysis

The data analysis was conducted sub-group blind (i.e. the trial statistician did not know which group was the intervention and which the control). An intention-to-treat (ITT) approach was used for the primary efficacy analysis, analysing patients according to the group to which they were randomized irrespective of whether or not they carried out the SR exercises. A *t*-test, with 95% confidence interval was initially used to test for a difference in mean STI scores between the two groups. A general linear model was then constructed with the STI baseline score, severity of reported numbness and difficulty grasping at baseline as covariates (i.e. adjusted for baseline prognostic variables pre-specified in the statistical analysis plan), to estimate an ‘adjusted’ between-group mean difference, with 95% confidence interval. A similar approach was used for secondary endpoints.

A secondary analysis utilized complier-averaged-causal-effects (CACE) approach (Hewitt et al., 2006) to estimate the effect size in those deemed to be adherent to SR. Adherence data in the intervention group was based on 38 diaries that were returned. ‘Compliers’ (i.e. those adherent) were defined by clinical expert members of the Trial Steering Committee for the Statistical Analysis Plan, with a patient deemed to be adherent if SR exercises were undertaken at least once a day, for at least 5 days out of 7 consecutive in a week and over at least three consecutive weeks. For a sensitivity analysis the threshold was raised to a 6-week duration, with the same day and week frequency.

### Data management

A trial specific Microsoft SQL (structured query language) database was set up by the data manager at the Norwich Clinical Trials Unit, held on a secure server and accessed through password by the CI and two research associates. All data entry from paper clinical record forms was doubled-checked by a member of the research team prior to data lock.

## Results

### Trial participants

**Figure 1.**
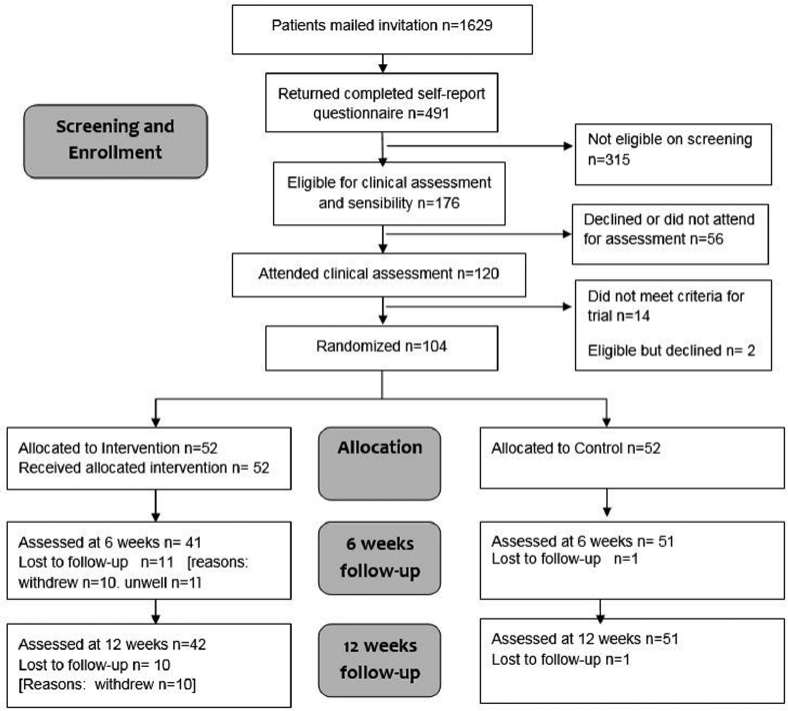
CONSORT diagram.

Between February 2014 and February 2015, 1629 patients were mailed an invitation and screening questionnaire. Figure 1 shows the number of patients who met eligibility at the screening and clinical assessment stage and the flow of randomized patients through the trial, in accordance with the CONSORT statement (Schulz et al., 2010).

A total of 120 patients who reported at least mild numbness and mild difficulty with grasping objects attended for clinical assessment of hand sensibility. Of these, 106 (88%) were found to have sensory impairment on at least two out of three tests. A total of 104 patients consented and were randomly assigned to the intervention (*n* = 52) or control group (*n* = 52). Baseline characteristics are summarized in Table 1.

**Table 1.** Baseline characteristics.

		Intervention (*n* = 52)	Control (*n* = 52)
Age mean (SD), years		71.8 (12.0)	70.8 (12.3)
Sex male/female		21 (40%)/31 (60%)	29 (56%)/23 (44%)
Dominant hand affected		29 (56%)	31 (60%)
Months since surgery, mean (range)		28.7 (11 to 106)	30.5 (13 to 73)
Self-reported numbness	Mild Moderate Severe Very severe	21 (40%) 22 (42%) 9 (17%) 0	24 (46%) 21 (40%) 7 (13%) 0
Self-reported difficulty grasping	Mild Moderate Severe Very severe	20 (38%) 19 (37%) 12 (23%) 1 (2%)	25 (48%) 17 (33%) 8 (15%) 2 (4%)
Previous injury or fracture		4 (8%)	4 (8%)
Past stroke		3 (6%)	3 (6%)
Other co-morbidity affecting the hand		18 (35%)	19 (37%)
Shape-Texture Identification (0–6)		3.6 (1.7)	3.4 (1.7)
Touch threshold (WEST) (0–4)		2.3 (0.65)	2.5 (0.63)
Locognosia (0–56)		42.7 (7.6)	42.6 (8.3)
Moberg test (seconds)		55.4 (38.3)	49.7 (35.9)
MHQ – overall function		55.7 (13.4)	55.2 (15.2)
MHQ – ADL affected hand		61.7 (22.0)	64.8 (23.5)
MHQ – ADL both hands		62.3 (21.0)	60.1 (22.7)
MHQ – work		64.2 (19.0)	60.6 (24.4)

ADL: activities of daily living; MHQ: Michigan Hand Questionnaire; SD: standard deviation; WEST: Weinstein Enhanced Sensory Test.

Ten patients withdrew, all from the intervention group (9.6%), and one patient was lost to follow-up in the control group. One patient in the intervention group missed the 6-week follow-up due to illness, but attended assessment at 12 weeks. Outcomes at 12 weeks were, therefore, obtained for 93 participants (89.4%). Patients who withdrew or were lost to follow-up tended to have poorer sensory scores and greater functional disability. Reasons for withdrawal included deteriorating general health (*n* = 2), a perceived lack of benefit to the trial (*n* = 2), frustration caused by perceived lack of improvement despite undertaking exercises (*n* = 2) and finding the exercises boring or difficult to fit around work (*n* = 1). Patients who expressed a wish to withdraw were encouraged by research staff to discontinue the intervention only and to remain in the trial, however most declined as they felt they were no longer of benefit to the trial.

### Outcomes

One report of an adverse event was received, which may have been related to the intervention. The patient had ongoing pain in their hand and arm and reported that this seemed to be aggravated by the exercises. The patient was advised to discontinue the intervention, the issue was discussed by the Trial Management Group and Steering Committee and no further action was taken. The patient completed the 6- and 12-week follow-up and was included in the analysis as per allocated arm (treatment group).

Unblinding of assessors occurred in 11 cases at the 6-week assessment and a further seven cases at the 12-week assessment. Most cases of unblinding were in the intervention group (nine at 6 weeks, five at 12 weeks). In some instances this occurred when the research staff who were also assessors contacted patients to remind them of their appointment, but in most cases unblinding happened during the assessment when patients noted that they had improved on objective tests and would attribute this to the ‘exercises’. Assessors were asked to guess each patient’s group allocation after the final 12-week assessment. Excluding those who had unblinded the assessor, the assessors correctly guessed 54% to be in the intervention group and 59% in the control group.

Tables 2 and 3 show the results of the ITT efficacy analysis at 6 and 12 weeks for the primary and secondary outcomes. There were no statistically significant differences between the groups at 6 weeks on the STI test (adjusted mean difference −0.47, 95% CI −1.09 to 0.14, *p* = 0.129) or at 12 weeks (adjusted mean difference −0.18, 95% CI −0.78 to 0.42, *p* = 0.549). Similarly differences between groups on secondary outcomes were not statistically significant with the exception of the patient-reported subscales of the MHQ. These included overall hand function (adjusted mean difference −6.61, 95% CI −11.0 to −2.2, *p* = 0.003), ADL affected hand (adjusted mean difference −6.36, 95% CI −12.5 to −0.3, *p* = 0.040) and bilateral ADL (adjusted mean difference −6.04, 95% CI −11.6 to −0.5, *p* = 0.032). The significant difference in the MHQ was retained in two subscales by 12 weeks (overall hand function and bilateral ADL).

**Table 2.** Efficacy analysis: ITT analysis at 6 weeks.

	Control (*n* = 51)	Intervention (*n* = 41)	Unadjusted mean difference (95% CI)	Adjusted mean difference^1^ (95% CI)
STI Score	3.63 (1.79)	4.20 (1.85)	−0.57 (−1.32 to 0.19) *p* = 0.139	−0.47 (−1.09 to 0.143) *p* = 0.129
Locognosia Score	44.8 (6.66)	44.9 (8.71)	−0.09 (−3.28 to 3.09) *p* = 0.954	−0.27 (−2.16 to 1.62) *p* = 0.774
LogMoberg Score	3.72 (0.54)	3.66 (0.59)	0.06 (−0.17 to 0.30) *p* = 0.601	0.02 (−0.12 to 0.16) *p* = 0.776
WEST Score	2.52 (0.70)	2.73 (0.73)	−0.21 (−0.51 to 0.084) *p* = 0.159	−0.07 (−0.32 to 0.18) *p* = 0.555
MHQ – Overall Function	52.0 (16.4)	60.2 (13.3)	−8.28 (−14.6 to −2.0) *p* = 0.010	**−6.61** (−11.0 to −2.2) *p* = 0.003
MHQ – ADL affected hand	60.8 (24.7)	70.7 (24.8)	−9.95 (−20.2 to 0.35) *p* = 0.058	**−6.36** (−12.5 to −0.3) *p* = 0.040
MHQ – ADL both hands	63.8 (22.6)	70.4 (22.9)	−6.60 (−16.1 to 2.9) *p* = 0.170	**−6.04** (−11.6 to −0.5) *p* = 0.032
MHQ – work	62.4 (20.6)	69.4 (24.7)	−7.04 (−16.4 to 2.3) *p* = 0.140	−3.83 (−2.5 to 10.2) *p* = 0.231

1 Adjusted for age, baseline score, difficulty grasping and numbness at baseline. Significance below 5% in bold.

ADL: activities of daily living; CI: confidence interval; MHQ: Michigan Hand Questionnaire; STI: Shape-Texture Identification; WEST: Weinstein Enhanced Sensory Test.

**Table 3.** Efficacy analysis: ITT analysis at 12 weeks.

	Control (*n* = 51)	Intervention (*n* = 42)	Unadjusted mean difference (95% CI)	Adjusted mean difference^1^ (95% CI)
STI Score	4.02 (1.59)	4.19 (1.78)	−0.17 (−0.87 to 0.53) *p* = 0.627	−0.18 (−0.78 to 0.42) *p* = 0.549
Locognosia Score	46.4 (7.41)	46.5 (7.47)	−0.14 (−3.22 to 2.94) *p* = 0.930	−0.28 (−2.23 to 1.68) *p* = 0.777
LogMoberg Score	3.62 (0.53)	3.46 (0.53)	0.16 (−0.06 to 0.38) *p* = 0.621	0.11 (−0.02 to 0.25) *p* = 0.084
WEST Score	2.73 (0.69)	2.87 (0.62)	−0.14 (−0.42 to 0.13) *p* = 0.297	−0.02 (−0.26 to 0.213) *p* = 0.853
MHQ – Overall Function	55.3 (15.1)	63.3 (17.2)	−8.04 (−14.7 to −1.4) *p* = 0.019	−**5.87** (−10.8 to −1.0) *p* = 0.019
MHQ – ADL Affected Hand	63.0 (23.9)	73.1 (25.1)	−10.1 (−20.2 to 0.0) *p* = 0.051	−6.56 (−14.1 to 1.0) *p* = 0.085
MHQ – ADL Both Hands	65.1 (22.4)	73.8 (23.0)	−8.68 (−18.1 to 0.7) *p* = 0.070	**−7.24** (−13.3 to −1.2) ***p*** = 0.019
MHQ - Work	67.0 (20.2)	75.1 (20.9)	−8.16 (−16.6 to 0.3) *p* = 0.059	−4.50 (−10.1 to 1.1) *p* = 0.110

1 Adjusted for age, baseline score, difficulty grasping and numbness at baseline. Significance below 5% highlighted in bold.

ADL: activities of daily living; CI: confidence interval; MHQ: Michigan Hand Questionnaire; STI: Shape-Texture Identification; WEST: Weinstein Enhanced Sensory Test.

Adherence diaries were received from 38 of the 42 patients who completed all follow-up. A total of 17 patients (45%) indicated that they continued to do the SR after 6 weeks, mostly because they felt it was beneficial and hoped to see further improvement. They also found it easy and painless to do. Several patients abandoned the exercises within only a few days, while one patient undertook the exercises much more frequently and for longer periods resulting in a right skewed distribution, therefore medians and interquartile range (IQR) were used to summarize the data (Table 4).

**Table 4.** Summary statistics for 6 week adherence diary (*n* = 38).

	Recommended minimum	Diary reported adherence		
		Median	Interquartile range	Minimum–maximum
Number of sessions per day	3	2	1–2	0 to 5
Total number of sessions over 6 week period (42 days)	126	102	55–115	7 to 210
Average total time per day (minutes)	15	14	11.75–20	2 to 127
Total duration over 6 weeks (minutes)	630	598	410–842	42 to 5246

For the secondary CACE analysis, defining ‘compliance’ on 3-week adherence to exercise, 28 patients (74%) in the intervention group were deemed ‘compliers’, i.e. they reported the minimum dose of treatment. A sensitivity analysis using a 6-week adherence threshold was performed in which 22 (58%) were deemed ‘compliers’ (Table 5). The estimates of treatment effect on the STI test at 6 weeks and 12 weeks are larger relative to the ITT analysis but are not statistically significant.

**Table 5.** Secondary CACE analysis.

	STI at 6 weeks Mean difference (95% CI)	STI at 12 weeks Mean difference (95% CI)
Adherence threshold 3 weeks minimum	−0.77 (−1.81 to 0.28)	−0.23 (−1.18 to 0.72)
Adherence threshold 6 weeks minimum	−0.97 (−2.28 to 0.35)	−0.29 (−1.48 to 0.96)

## Discussion

In this pragmatic, randomized, controlled trial we found that a 6-week unsupervised home SR programme was not clinically effective in improving objective measures of hand sensibility in the short-term. The size of effect on the primary outcome (STI) at 6 weeks is estimated at around half a point, but with an upper confidence limit that includes the size deemed clinically important and was used in the sample size calculation (i.e. 1.09 units) (Jerosch-Herold, 2003). The intervention did have a statistically significant effect on patient-reported hand function (MHQ subscales for overall hand function and ADL), however this must be interpreted with caution. First, those patients who withdrew (19%) from the intervention group and were therefore not included in the 6 and 12 weeks analysis, had poorer sensory and functional scores at baseline. Therefore, the better score seen in the intervention group may simply be a bias from excluding those in the group with the worst scores. Second, the mean difference is below what might be considered clinically important (Shauver and Chung, 2009). Third, the MHQ was patient-reported and therefore not blinded, which may have created a bias in response.

The secondary CACE analysis found that considering those adherent with the recommended exercise frequency and duration did not have a significant effect on the primary outcome. The effect was increased relative to that estimated with the ITT analysis, especially when using the stricter definition of adherence (6 weeks), but this is to be expected when using a CACE analysis. Moreover, the lower confidence limit is >2, a difference that would be deemed clinically important for the STI test. The advantage of using CACE analysis, compared with per protocol analysis, is that it maintains comparability of groups achieved through randomization, assuming that the probability of non-compliance with the intervention would be the same in the control and intervention group (Hewitt et al., 2006). Both approaches will result in an estimated treatment effect larger than that estimated from the ITT approach.

Previous indications that SR may be beneficial observed in a pilot study (Jerosch-Herold et al., 2012) were not substantiated in this definitive trial. This was despite the intervention and follow-up period in the present trial being longer (6 and 12 weeks compared with the 4 and 8 weeks in the pilot trial) and improvements being made to the SR programme to facilitate adherence. The eligibility criteria in the present trial specified that patients had to be at least 12 months since surgery as opposed to 6 months in the pilot trial. The mean time since surgery was much longer in the present trial, which may account for a more chronic sensory impairment that could also be more resistant to treatment.

One possible reason for the lack of effect is that ‘dose’ and duration of the intervention were too low for learning to take effect. SR requires short but frequent practice in tactile discrimination tasks. In this study patients were given an unsupervised home programme. The exercises may not have been enacted as intended and there was no interaction with the study team between baseline and 6-week follow-up to check on progress and understanding. Such interaction may also have helped mitigate the attrition in the intervention group. Second, we cannot rule out the possibility that patients in the control group may have changed the way they used their hands, or sought information about SR through the internet. The sensory tests may have resulted in patients attending more to tasks requiring touch perception and discrimination. Both groups showed a small improvement in sensory scores over time. This could also be due to a practice effect from repeated sensory testing.

Another plausible reason for the lack of observed effect may be that the intervention was too generic and that a ‘one size fits all’ approach does not work. SR was standardized, but some patients noted in their diaries that they found certain exercises, such as the object identification, too easy. We did emphasize the need for patients to self-monitor and replace the objects with ones that were smaller and increasingly complex. Some patient diaries indicated that they replaced objects and also began to transfer the principles of SR into their everyday activities and hobbies by trying to undertake tasks requiring fine dexterity with their eyes closed, attending to the feel of the surface or object and using vision only intermittently. These patients also reported greater benefit from the intervention and continued to practise SR after the initial 6 weeks. However, it is also evident from the diary comments that for some the SR programme was too easy or boring and those patients lost motivation, some withdrawing from the intervention or the trial altogether.

Finally, the mean age of the trial participants was relatively high (>70 years). Although cognitive function declines with age, the ability to learn new skills (neural plasticity) remains (Cai et al., 2014; Leung et al., 2015). The way in which the exercises were enacted may have varied depending more on the person’s cognitive ability, motivation and activity levels rather than age per se.

This trial posed several challenges. First, to identify patients with sensory impairment after carpal tunnel compression. Patients are not routinely assessed after surgery and therefore chronic sensory impairment is rarely documented in primary care records. However, the patient self-report did prove to be highly sensitive in identifying those who have sensory deficits on objective testing (88%). The second challenge was the high attrition observed in the intervention group. Even for those who did remain in the trial, adherence to the intervention was suboptimal in 42% of patients allocated to the intervention. Reasons for withdrawal and non-adherence appear to be a combination of patient and intervention-related factors. First, the intervention may have not been a good fit to the patients’ specific problems thus resulting in early non-adherence. Furthermore, even among those who intended to comply or who were partial compliers, many forgot or were unable to find the time in their daily routine to do the exercises. SR requires patients to relearn the way they attend to their touch sense. Compensatory behaviours such as relying on vision or using the other hand due to poor sensibility may need to be ‘unlearned’. Factors such as patient self-efficacy, motivation and opportunity to engage with the exercises may play an important role in whether SR is seen as a worthwhile behaviour to learn. Behaviour change taxonomy (Michie et al., 2015), which has been applied in the context of public health interventions such as promoting increased physical activity, may offer a useful framework for future sensory relearning intervention development.

### Strengths and limitations

Strengths of this trial include the use of objective standardized measures of hand sensibility, and an adequately powered sample size. The intervention used in this trial, including the materials and instructions, were standardized and therefore replicable. In order to maximize treatment fidelity, the same person (CI) delivered the intervention to all patients.

There are also several limitations. Although loss to follow-up was only 10% for the whole cohort, attrition was much greater in the intervention group and may have biased the outcome. While assessor blinding was used, there were several instances of unblinding. The most likely bias arising from unblinded assessment is that it favours the experimental intervention and leads to an overestimate of effect (Poolman et al., 2007), however this was not observed in our trial. Finally, adherence with SR was patient reported and could not be independently verified.

### Conclusions and implications

Identification of chronic sensory impairment after carpal tunnel decompression and targeting of therapies to improve functional sensibility and hand function in this population may require alternative strategies. At present there is no evidence to support the use of unsupervised SR as an intervention for patients with tactile sensory and functional deficits after carpal tunnel decompression. Further research is needed on how SR programmes can be designed that are practicable, affordable and sufficiently challenging to maximize adherence. This may require more individually tailored exercises to address specific sensory deficits, more therapist supervised sessions or at least frequent monitoring of patient progress to grade and progress the complexity of the therapy programme. There is also the potential to use multimedia, including online video instructions demonstrating the exercises or development of smartphone applications to monitor SR practice, enhance motivation and provide positive reinforcement.
